# A non-classical presentation of scar endometriosis during pregnancy: Case report and review of literature

**DOI:** 10.5935/1518-0557.20210100

**Published:** 2022

**Authors:** Garima Sachdeva, PS Divyashree, N Shailaja

**Affiliations:** 1 Department of Reproductive Medicine, Milann, Bangalore, India

**Keywords:** endometriosis, pregnancy, fibrosis, scar, MRI, ultrasound, histopathology

## Abstract

Scar endometriosis is an uncommon condition in which endometrial tissue grows in a previous surgery wound site. The triad of scar endometriosis includes the history of caesarean section or any other gynecological surgery, cyclical waxing, and waning pain accompanied by the patient's menstrual cycle and a tumor inside/near the scar site. Here, we present a case report in which the patient presented with endometriosis at the previous caesarean section scar site. She had classical clinical and imaging characteristics for scar endometriosis. However, histopathology was not diagnostic for the same. The pathogenesis, presentation, diagnosis, and management are discussed briefly. The diagnosis of scar endometriosis is based on clinical presentation and imaging. Fibrosis on histopathology is an important component of endometriosis that cannot be overlooked.

## INTRODUCTION

Scar endometriosis is an uncommon presentation of extra-pelvic endometriosis and presents with pain and swelling at the previous caesarean section scar site that increases during menstruation. Its reported incidence is around 0.03-0.47% (Chang *et al*., 2009). The proposed mechanism behind the development of scar endometriosis is the iatrogenic transplantation of the endometriotic implants/stem cells into the surgical area with progression to endometriosis anywhere between three months and 12 years of the previous abdominal/pelvic surgery (Witz, 1999). The transplanted endometrial tissue may either proliferate under the influence of estrogen or can cause metaplasia of the surrounding tissue resulting in the formation of endometrioma (Witz, 1999). Other mechanisms include lymphatic or hematogenous dissemination of endometrial cells that can grow into the endometrioma (Witz, 1999).

Scar endometriosis is usually restricted to the skin and subcutaneous tissue, and very rarely involves fascia, muscles, and pelvic organs. Here, we present a case report in which the patient had classical clinical and imaging characteristics for scar endometriosis. However, histopathology was not diagnostic for the same. Whether the histopathology findings were due to pregnancy-related changes in the endometriosis tissue is a matter of debate and further research.

## CASE REPORT

A 33-year P1L1 woman came to our clinic last year complaining of pain and swelling at the site of a caesarean section scar that had been persisting for 6 months. Pain was dull aching/stretching type and increased during menstruation. There was no change in the color of the skin over the mass or bleeding from the mass. The patient had undergone an uneventful lower segment caesarean section (LSCS) five years prior. She did not report any other significant past medical or surgical event to.

**Figure 1:**
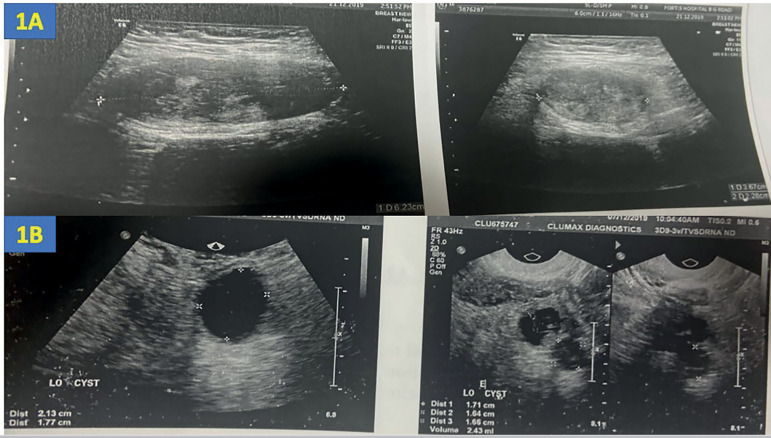
1A: Ultrasound scan illustrating a well-defined hypoechoic lesion with smooth margins in the muscular plane of the suprapubic region measuring 6.23cm x 3.67cm x 2.28cm. 1B: Ultrasound scan showing an endometrial cyst measuring 1.71cm x 1.66cm x 1.64cm and a clear cyst measuring 2.13cm x 1.77cm in the left ovary.

**Figure 2:**
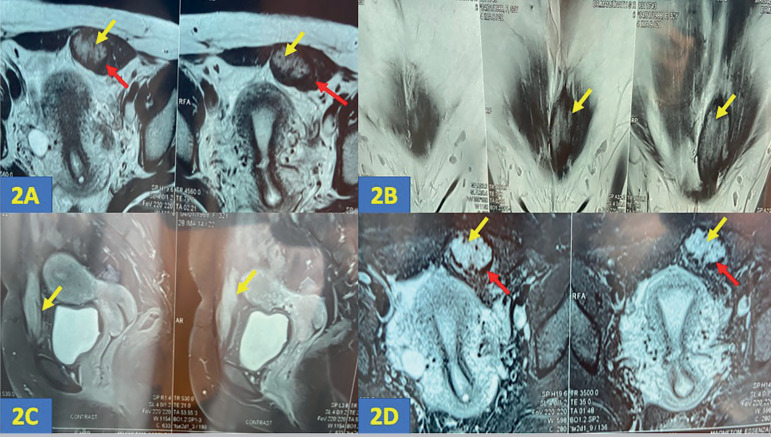
2A (axial T2WI), 2B (coronal T2WI), 2C (T1WI-post contrast), 2D (STIR axial): the yellow arrows in these images reveal a relatively well defined nodular lesion in the supra-pubic region within the bulky left rectus abdominis muscle, measuring 5.8cm x 3.3cm x 2.5cm. The lesion appears heterogeneously hyperintense on T2WI and STIR, showing a peripheral hypointense rim due to hemosiderin deposition (red arrow- 2A, 2D) with post contrast enhancement (2C).

Examination revealed a 6cm x 4cm, tender, and immobile subcutaneous mass beneath the previous caesarean section scar site. Her body mass index (BMI) was 25.7kg/m^2^. An ultrasound scan revealed a well-defined hypoechoic and heterogeneous mass with internal echoes with smooth margins in the muscular plane of the suprapubic region measuring 6.23cm x 3.67cm x 2.28cm (Figure 1A). An endometrial cyst measuring 1.71cm x 1.66cm x 1.64cm and a clear cyst measuring 2.13cm x 1.77cm were found in her left ovary (Figure 1B). Her right ovary and uterus were normal. Ultrasound findings were suggestive of scar endometriosis. Magnetic resonance imaging (MRI) was performed to confirm the diagnosis. The scans revealed a relatively well-defined nodular lesion in the supra-pubic region within the bulky left rectus abdominis muscle, measuring 5.8cm x 3.3cm x 2.5cm. The lesion appeared heterogeneously hyperintense on T2WI (T2 weighted images) and STIR (short tau inversion recovery), showing a peripheral hypointense rim due to hemosiderin deposition (red arrow- Figure 2A, 2D) with post-contrast enhancement (Figure 2C).

**Figure 3:**
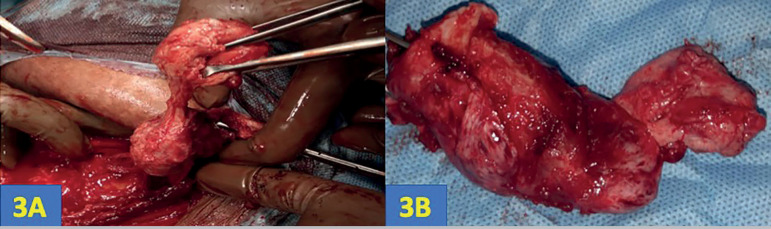
3A: Wide excision of scar endometriotic tissue. Figure 3B: Post-excision sample of scar endometriotic tissue sent for histopathology.

**Figure 4:**
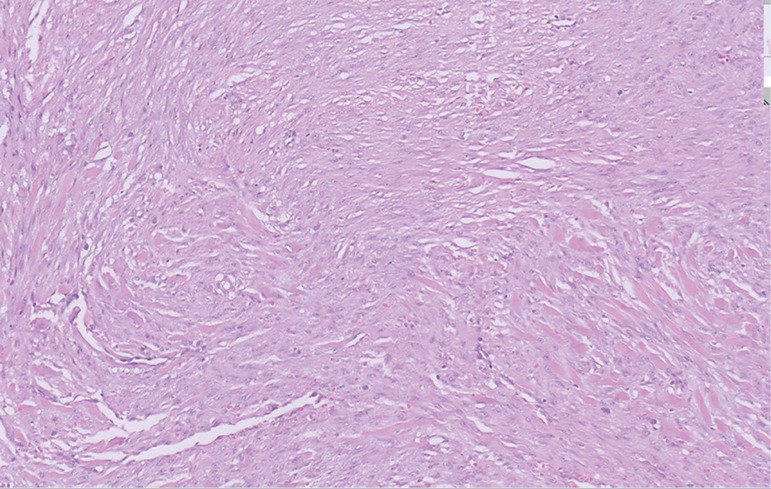
Histopathology showing fibro-collagenous tissue with striated muscle deeper down. There are vague fascicles of spindle cells with fusiform nuclei and scanty cytoplasm, in a densely collagenous stroma. Few extravasated red cells were seen in areas of hemorrhage. No endometrial glands seen.

Although the patient was offered surgery on account of the characteristic history and radiology findings, she preferred to undergo medical management. Hence, she was treated with dienogest tablets 2mg daily for 3 months, with which her symptoms improved and the size of the mass reduced (4.7cm x 3.5cm x 2.5cm). Since the patient was anxious to conceive, she was advised to discontinue treatment with dienogest and to try naturally for pregnancy. She conceived naturally after 4 months of stopping dienogest. She did not develop any symptoms during pregnancy, but could still feel the mass, as also seen on interval growth scans. Her pregnancy was uneventful and she underwent elective lower segment caesarean section with bilateral tubal ligation with wide excision of scar endometriotic tissue at 38±5weeks (Figure 3). Histopathology revealed fibro-collagenous tissue with striated muscle deeper down. There were vague fascicles of spindle cells with fusiform nuclei and scanty cytoplasm, in a densely collagenous stroma. Few extravasated red cells were seen in areas of hemorrhage. No endometrial glands were seen (Figure 4). The patient is in our follow-up program and has not reported recurrence of symptoms or mass.

## DISCUSSION

Cesarean section appears to be an important risk factor of scar endometriosis, as it exposes a large number of endometriotic cells and these cells get entrapped into the wound (Wang *et al*., 2003). Moreover, amniotic fluid and excessive blood loss exacerbate the separation of these active cells and provide a rich nourishing environment encouraging the growth of endometrial tissue in the wound (Wang *et al*., 2003). Obesity is an additional risk factor as it provides a wide surface area for entrapment of endometrial tissue (Uçar *et al*., 2015).

Esquivel-Estrada *et al*. (2004) described the triad seen in cases of scar endometriosis, which comprises history of caesarean section or any other gynecological surgery, cyclical waxing, and waning pain accompanied by the patient's menstrual cycle, with a tumor inside/near the scar site as the clinical diagnostic sign for scar endometriosis. Our patient fulfilled all three criteria.

Magnetic resonance imaging (MRI) and ultrasonography (USG) are the most commonly used non-invasive imaging modalities for endometriosis (Nisenblat *et al*., 2016). The sensitivity and specificity of USG in diagnosing endometriomas is 65% and 95% respectively (Nisenblat *et al*., 2016). The sensitivity and specificity of MRI in diagnosing endometriomas is 90-92% and 91-98%, respectively (Kinkel *et al*., 2006). In our case, both USG and MRI added to the clinical diagnosis of the disease.

Histopathologic diagnosis can be made based on the presence of two out of three features: 1) presence of endometrial type glands, 2) endometrial stroma (often contains fine capillary network, long-standing cases may show fibrosis or decidual change, myxoid change), and 3) evidence of chronic hemorrhage (hemosiderin-laden/foamy macrophages) (Pathan *et al*., 2005). The histopathology findings were not classical in our case. However, evidence of fibrosis was noted, which is a characteristic feature of long-standing stromal endometriosis (Vigano *et al*., 2018). Vigano *et al*. (2018) have suggested the inclusion of fibrosis as an important part of the definition of endometriosis, not only for diagnostic but also for treatment purposes. In fact, 40% of the cases of ovarian endometriosis are devoid of endometrial epithelium, and in most of these cases, only the inner cyst wall demonstrates only fibrotic changes (Muzii *et al*., 2007). Moreover, pelvic adhesions classically seen in endometriosis are devoid of any endometrial components (Somigliana *et al*., 2012). In our case, the only finding evidenced in the excised tissue was fibrosis. Typical endometrial glands were not seen in our patient possibly because of the pregnancy-associated regression of endometriosis (Leeners *et al*., 2018). The presence of endometrial tissue in such patients can be confirmed with immunohistochemistry staining for ER (estrogen receptor) and PR (progesterone receptor).

The various indicators pointing towards the diagnosis of scar endometriosis in the case reported herein include history of cyclical pain increasing during menstruation and presence of a mass (confirmed by ultrasound and MRI scan) at the previous cesarean section scar site. The resolution of symptoms and the decrease seen in the size of the mass after the introduction of dienogest along with the absence of symptoms during pregnancy point towards the diagnosis of scar endometriosis.

The first-line management of scar endometriosis is wide surgical excision of the mass (Vagholkar & Vagholkar, 2019). Medical management can be considered for symptom management, which includes treatment with oral contraceptive pills, progestins, dienogest, or gonadotropin releasing hormone agonists (Dunselman *et al*., 2014).

Since the symptoms were mild and the patient was planning for future pregnancy, she was treated medically with dienogest and surgical excision was deferred until the next caesarean section to prevent an additional surgery.

Several preventive measures have been proposed in the literature to minimize the iatrogenic transplantation of endometriotic tissue, such as thoroughly cleaning and irrigating the abdominal wound before closure (Uçar *et al*., 2015). Also, it is a common practice amongst obstetricians to clean the endometrial cavity with a moist/dry sponge after placental removal and before uterine closure. If the same mop is used, it can cause the inoculation of endometrial tissue onto the abdominal wound site (Wolf & Singh, 1989). Moreover, the suture material used to close the abdominal wall should not be re-used to close the abdominal wound (Uçar *et al*., 2015). The instruments and needles used in uterine wall closure should be replaced with new ones when the abdominal wall is sutured (Teng *et al*., 2008). Some studies have advocated that the closure of visceral and peritoneal layers might also help in the prevention of scar endometriosis (Chang *et al*., 2009).

## CONCLUSION

Scar endometriosis is an uncommon disease caused by iatrogenic inoculation of endometrial tissue onto the abdominal scar site. Caesarean section is one of the common causes of scar endometriosis and adequate measures should be taken to prevent this iatrogenic complication. It is important to include fibrosis in the definition of endometriosis. Wide surgical excision is the first-line treatment option for scar endometriosis. If scar endometriosis develops after caesarean section and the patient is having mild symptoms and has plans to get pregnant in the future, excision may be deferred until the next caesarean section.
